# Antimicrobial property of *Pichia pastoris*‐derived natto peptide against foodborne bacteria and its preservative potential to maintain pork quality during refrigerated storage

**DOI:** 10.1002/fsn3.2722

**Published:** 2022-01-08

**Authors:** Bin Dong, Cailing Yu, Yanjun Lin, Guowen Zhou, Chunlong Sun, Jun Wang, Tao Wu

**Affiliations:** ^1^ Shandong Provincial Engineering and Technology Research Center for Wild Plant Resources Development and Application of Yellow River Delta College of Biological and Environmental Engineering Binzhou University Binzhou China

**Keywords:** antimicrobial agents, antimicrobial peptides, antioxidant activity, natto peptide, pork, preservatives

## Abstract

Pork spoilage caused by foodborne bacteria contamination always leads to substantial economic loss in the meat industry. The toxicity and drug resistance of chemical preservatives have raised public concerns about their safety and stability. In this study, natto peptide from *Pichia pastoris* was prepared using DNA recombinant technology. It showed an excellent antibacterial effect against Gram‐positive and ‐negative bacteria, with minimum inhibitory concentrations (MICs) ranging from 6 to 30 μg/ml. Of note, natto peptide exhibited low cytotoxicity and hemolytic activity. The application of natto peptide on pork during refrigerated storage dramatically decreased the growth of *Staphylococcus* spp., *Escherichia* spp., and *Pseudomonas* spp. The bactericidal properties remained in force when natto peptide was used in pork models contaminated with artificial bacteria. Moreover, the application of natto peptide (90 μg/ml) inhibited the increase in pH variation and drip loss, decreased the generation of total volatile basic nitrogen (TVB‐N) and thiobarbituric acid reactive substances (TBARS), and maintained a high sensory quality score during pork storage. These results implied that *P. pastoris*‐derived natto peptide could extend the storage time of pork, and it has the potential to be a promising antiseptic biopreservative to replace chemical preservatives.

## Graphical abstract

The natto peptide could significantly extend the shelf life of fresh pork. The natto peptide maintained a high quality of fresh pork during storage at 4℃. The natto peptide had the potential to replace chemical preservatives during pork storage.

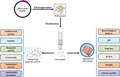

## 1 INTRODUCTION

Pork, which contains excellent animal proteins, minerals, and vitamins, is an important source of essential nutrients for people in daily life (Bohrer, 2017; Henchion et al., 2014). In recent years, consumers not only care about the types and sources of meat but also pay increasing attention to the quality and safety of meat for improvement in living standards and conceptual change. In general, the pork quality and safety are closely related to nutritional constituents, processing, and storage methods, which may affect the color, drip loss, and sensory characteristics. However, owing to the high content of protein and lipids in pork, once contaminated with microorganisms, such as *Escherichia coli*, *Staphylococcus aureus*, and *Pseudomonas aeruginosa*, pork and its products could easily spoil and cause substantial economic loss during processing, transportation, and storage (Gram et al., 2002; Zhou et al., 2010). Thus, microorganism pollution is a serious problem in pork preservation to maintain quality and safety. Various methods have been used in the pork preservation area, including physical and chemical strategies (Gogliettino et al., 2019; Lee et al., 2021; Papadochristopoulos et al., 2021), to inhibit the growth of foodborne microorganisms. Unfortunately, pork spoilage with microorganism pollution could still not be totally eliminated. Chemical preservatives, including sodium benzoate and potassium sorbate (Erickson & Doyle, 2017), have been used in food storage, such as beverages, meat, and cereal foods, for decades. However, the toxicity and drug resistance of chemical preservatives and abuse in their application have raised public concerns about their safety and stability (Eijlander et al., 2011).

An increasing number of researchers have focused their attention on the development of new kinds of antimicrobial agents. Antimicrobial peptides (AMPs), which are generally small‐molecular‐weight peptides containing 3–200 amino acids derived from a wide range of organisms, including prokaryotic and eukaryotic organisms, exhibited broad antimicrobial spectrum against bacteria, fungi, and parasites (Zasloff, 2002). Moreover, AMPs showed no drug resistance and possessed stable physical and chemical properties (Wang et al., 2019; Xu & Lai, 2015). Therefore, they have been recognized as promising antibiotic substitutes. Nisin, which is a typical AMP that exerts potent antimicrobial activity, has been used in food storage as a safe and efficient biopreservative all around the world for decades (Gharsallaoui et al., 2016).

Natto is a traditional Japanese fermented soybean product that contains numerous valuable nutrients, including vitamins, isoflavone, lecithin, and nattokinase (Berenjian et al., 2014). It is considered a healthy food. Nattokinase is a member of the subtilisin family, which is composed of 275 amino acids and possesses therapeutic potential for the treatment of thrombovascular diseases, hypertension, Alzheimer's disease, and vitreoretinal disorders (Dabbagh et al., 2014). In a previous study (Kitagawa et al., 2017), Shinichi Yokota's group identified a natto peptide consisting of 45 amino acid residues from natto extract. It shared 97.8% homology with the C‐terminal region of nattokinase produced by *Bacillus subtilis*. Furthermore, the natto‐derived peptide showed a narrow antimicrobial effect against *Streptococcus pneumoniae* and *B. subtilis* only. The predicted structural characteristics of the peptide were similar to those of cathelicidin family AMPs, which exhibited a broad antimicrobial spectrum (Margherita et al., 2002; Sang & Blecha, 2008). Meanwhile, the source of peptide is a healthy food with medical function. Therefore, natto peptide may be a promising antimicrobial agent that could be used in food preservation. DNA recombinant technology was used to solve the problems of extraction from natural products to improve the antimicrobial property and productivity of the peptide. *Pichia pastoris* is a classical expression host that produces recombinant proteins compared with prokaryotic hosts, such as *E. coli* and *B. subtilis* (Ahmad et al., 2014; Karbalaei et al., 2020). Many bioactive peptides have been produced in *P. pastoris* by the DNA recombinant technology, as for the advantages of posttranslational modifications such as protein folding and glycation (Karbalaei et al., 2020). Many AMPs have been reported to express *P. pastoris*, and their antimicrobial activity and spectrum improved compared with those of natural extracted products, including Mytichitin‐A (Meng et al., 2016), Padef (Meng et al., 2017), Hispidalin (Meng et al., 2019), and Lactolisterin BU (Dong et al., 2021).

This study aimed to improve the antimicrobial property of natto peptide by using DNA recombinant technology with *P. pastoris* system and analyze the antimicrobial effect of the purified natto peptide. The antimicrobial application of purified natto peptide in pork preservation was initially evaluated by investigating the growth of bacterial pathogens, pH, total volatile basic nitrogen (TVB‐N), thiobarbituric acid reactive substance (TBARS), and sensory evaluation of pork samples during storage.

## 2 MATERIALS AND METHODS

### 2.1 Strains, vectors, and reagents

Bacterial strains such as *B. subtilis* American Type Culture Collection (ATCC) 6633, *B. subtilis* AHU 1035, *Listeria monocytogenes* ATCC 221633, *L. monocytogenes* ATCC 19115, *L. monocytogenes* CMCC(B) 54002, *S. aureus* ATCC 43300, *S. aureus* ATCC 25923, *E. coli* ATCC 25922, *E*. *coli* ATCC 8739, *E. coli* O157:H7 ATCC 35150, *Enterobacter sakazakii* ATCC 29544, *Salmonella typhimurium* ATCC 14028, and *P. aeruginosa* ATCC 902 were purchased from the National Center for Medical Culture Collections. *P. pastoris* yeast strain X‐33, *E. coli* DH5α strain, and pPICZα‐A plasmid were purchased from Invitrogen Company. They were used for gene cloning and protein expression. All other chemicals were obtained from Solarbio, except those described.

### 2.2 Antimicrobial activity assays

#### 2.2.1 MIC

The antimicrobial effect of natto peptide was determined using microbroth dilution assay (Dong et al., 2018). In brief, various concentrations of the natto peptide (0–120 μg/ml) were incubated with bacterial cultures (6 × 10^5^ CFU/ml) for 14–16 h at 37°C to determine the minimum inhibitory concentration (MIC), which refers to no obvious colonies that occurred when the cultures were incubated overnight. Phosphate‐buffered saline (PBS) buffer and gentamicin were used as negative and positive controls. All experiments were performed three times.

#### 2.2.2 Time‐killing curves

Time‐killing curves were obtained as described previously to evaluate the bactericidal rates of natto peptide (Dong & Sun, 2021). Concisely, the natto peptide at final concentrations of 1×, 2×, and 4× MICs was incubated with *E. coli* O157, which was cultured to midlog phase. Then, the culture samples were harvested at different time intervals for colony counting. PBS buffer and gentamicin were used as negative and positive controls. All the experiments were performed in triplicate.

#### 2.2.3 Biofilm formation

Crystal violet staining method was performed as described previously to evaluate the biofilm inhibition effect of natto peptide (Chen et al., 2017), and *E. coli* O157 was used as the tested strain. Butyl paraben was used as positive control, and Mueller‐Hinton (MH) broth was used as blank control.

### 2.3 Hemolysis, cytotoxicity, and stability of peptides

#### 2.3.1 Hemolysis

The hemolytic effect of natto peptide against rabbit erythrocytes was investigated, as described previously (Liu et al., 2020). PBS and 1% Triton X‐100 were used as blank and positive controls, respectively. In brief, blood cells were initially mixed with anticoagulant dipotassium EDTA, the erythrocytes were isolated by centrifugation at 1500*g* for 15 min and washed three times with PBS buffer. Then, the erythrocytes were resuspended with PBS at a ratio of 1:20 and incubated at 37°C with various concentrations of the natto peptide (0–200 μg/ml) for 1 h. After centrifugation at 1500*g* for 10 min at room temperature, the supernatants were transferred to a new 96‐well microplate to determine the absorbance value at 540 nm by using a microplate reader (HBS‐1096A; Detie). All assays were performed three times.

#### 2.3.2 Cytotoxicity

The cytotoxicity of natto peptide toward RAW264.7 cells was determined by the methyl‐thiazol‐diphenyltetrazolium (MTT) method, as described in our previous study (Xue et al., 2020). Briefly, the RAW264.7 cells were cultured in Rosewell Park Memorial Institute (RPMI)‐1640 medium containing 10% fetal bovine serum (FBS), 1% penicillin, and 1% streptomycin at 37°C in a 5% CO_2_ atmosphere environment. The diluted natto peptide with different concentrations (0–200 μg/ml) was incubated with RAW264.7 cells in 96‐well plates at 37°C in a 5% CO_2_ atmosphere. Then, the MTT solutions (1 mg/ml) were added in the cell suspensions for another 4 h of incubation in the dark at 37°C. After discarding the supernatants, the formed formazan crystals were resolved with dimethyl sulfoxide (DMSO) and the absorbance value at 570 nm was determined by using a microplate reader (HBS‐1096A; Detie). The cell viability was calculated as the following: cell viability (%) = OD sample/OD control × 100%. All the experiments were performed three times.

#### 2.3.3 Stability

Inhibition‐zone assay was used to determine the stability of natto peptide against *E. coli* O157, as described in a previous study (Dong & Sun, 2021). In brief, 6 μg/ml of natto peptide with different treatments, including dissolution in diverse pH (pH = 2, 4, 6, 8, and 10) or salinity (50, 100, 200, 300, 400, and 500 mM) buffers; incubation at various temperatures (4, 25, 37, 65, and 90°C); and digestion with papain, pepsin, trypsin, and proteinase K solutions, was used to determine the pH, salt, and thermal and protease stabilities. Untreated peptides and PBS were used as positive and negative controls, respectively. All experiments were conducted three times.

### 2.4 Antioxidant‐activity assay

The antioxidant effect of natto peptide was determined by investigating the scavenging capacities of 2,2′‐diphenyl‐1‐picrylhydrazyl (DPPH), 2,2′‐azino‐bis(3‐ethylbenzothiazoline‐6‐sulfonic acid) (ABTS^+^), hydroxyl and superoxide anion free radicals, as described previously (Dong et al., 2020). For the DPPH assay, the natto peptide was incubated with DPPH buffer which was dissolved in 95% methanol in a dark environment for 30 min, then the absorbance value at 517 nm was measured by using a microplate reader. In the ABTS^+^ assay, the natto peptide was incubated with an ABTS^+^ working solution in dark for 10 min, then the absorbance value at 734 nm was determined by using a microplate reader. Moreover, the ABTS^+^ working solution was prepared by using potassium persulfate solution (2.6 mM) to dilute the ABTS^+^ stock solution (7 mM) at a ratio of 1:1 and incubated in dark for 12 h before determination. The hydroxyl and superoxide anion radical scavenging abilities were determined by using a Hydroxyl Free Radical Scavenging Capacity Assay Kit (BC1325; Solarbio) and a Superoxide Anion Detection Kit (BC1290; Solarbio), according to the manufacturer's instructions. All determinations were repeated three times.

### 2.5 Application of natto peptide in pork storage

#### 2.5.1 Pork preparation and treatment with natto peptide

Fresh‐pork legs were bought from a local butcher (Binzhou, China) 24 h postmortem. The connective tissue and fat of the pork were removed before cutting into even slices (5 ± 0.1 g). Afterwards, the slices were soaked in 0, 30, 60, and 90 μg/ml of natto peptide for 20 min. Then, the slices were drained and placed in sterilized polyvinyl chloride trays, sealed using a polyethylene film, and stored at 4°C in a refrigerator for 8 days. Meanwhile, 50 μg/ml of nisin and 100 μg/ml of butyl paraben were used as positive controls to analyze the aseptic effects of natto peptide, including microbial growth, TVB‐N, lipid oxidation, pH, drip loss, and sensory quality during storage.

#### 2.5.2 Microbial growth analysis

The microbial growth of pork was determined by colony count, as described previously (Meng et al., 2021). In brief, after the pork sample (5 g) was homogenized with 0.9% sterilized saline buffer (25 ml), 100 μl of the 10× diluent was spread on plate‐count agar, mannitol‐salt agar, violet red bile agar, and centrimide (CN) agar, and incubated at a suitable temperature for 48 h. All experiments were performed three times.

#### 2.5.3 Microbiological‐challenge tests

*Escherichia coli* O157 ATCC 35150, *S. aureus* ATCC 25923, and *P. aeruginosa* ATCC 902 were used to pollute the fresh pork and construct a pork spoilage model, which was used to further evaluate the antimicrobial effect of natto peptide. Five grams of pork samples was inoculated with 100 μl of bacteria (2.0 × 10^5^ CFU (colony‐forming units)/piece) by infusion before the natto peptide was coated on the samples. Then, the pork samples were placed in sterilized polyvinyl chloride trays, sealed using a polyethylene film, and stored at 37°C for 24 h. Thereafter, 5 g of pork sample was homogenized with 0.9% sterilized saline buffer (25 ml), and 100 μl of the 10× diluent was spread on the corresponding agar and incubated at 37°C for 48 h. The bacterial colony count was illustrated as logarithms of colony‐forming units per gram (Log CFU/g). All assays were performed three times.

#### 2.5.4 Measurement of pH and drip loss

The pH values of the pork were measured in accordance with the National Standard of People's Republic of China (GB 5009.237‐2016) by using a pH meter (REX PHSJ‐5, Shanghai, China). Drip loss (%) was calculated using the following formula: drip loss (%) = (initial weight of pork − final weight of pork)/initial weight of pork × 100%. All assays were performed in triplicate.

#### 2.5.5 Measurement of TVB‐N

The TVB‐N value of pork samples was determined in accordance with the National Standard of People's Republic of China (GB 5009.237‐2016) by using Kjeldahl assay. In brief, 5 g of pork sample was completely homogenized with 25 ml of distilled water for 30 min. After filtering with a filter paper, the homogenate was mixed with 10 g/L of MgO at a ratio of 1:1 (v/v) and distilled. Then, the distillate was resolved in an equal volume of boric acid, which contained 0.1% methyl red and 0.1% bromocresol green. Finally, hydrochloric acid (0.01 mol/L) was used to titrate the solution and determine the TVB‐N value. All assays were performed in triplicate.

#### 2.5.6 TBARS assay

Thiobarbituric acid reactive substances assay was performed to investigate the process of lipid oxidation in pork by determining the malondialdehyde (MDA) product content. Concisely, 5 grams of pork sample was homogenized with 50 ml of 7.5% trichloroacetic acid buffer before filtering with a filter paper. Then, 5 ml of the filtered buffer was reacted with an equal volume of 2‐thiobarbituric acid (0.22 mol/L) at 100°C for 15 min and cooled to room temperature. Afterwards, the optical density at 532 nm of the reactant was measured using an ultraviolet‐visible (UV‐Vis) spectrophotometer (Nanjing Feile Instrument Co., Ltd.). The TBARS value was determined using the standard curve of 1,1,3,3‐tetramethoxypropane and described as milligram (mg) MDA equivalents per 100 g of pork. All assays were performed in triplicate.

#### 2.5.7 Sensory quality

Sensory quality, including color, odor, texture, and overall acceptance, was investigated using quality index analysis, as described previously (Dong et al., 2021). Ten experienced evaluators were selected to train and assess the sensory quality of pork samples in individual chambers, and the samples were scored on a scale of 1–10 (8–10 = excellent and highly acceptable, 6–8 = good and acceptable, 4–6 = poor and unacceptable, and 0–4 = very poor and very unacceptable).

### 2.6 Statistical analysis

All results were analyzed using GraphPad Prism 7.0 (GraphPad Software) by one‐way analysis of variance (ANOVA) and Duncan's multiple range test. Meanwhile, all data in the manuscript are expressed as mean ± standard derivation, and the results were defined as statistically different when *p* < .05.

## 3 RESULTS

### 3.1 Antimicrobial property of natto peptide

#### 3.1.1 MIC of natto peptide

The MIC of natto peptide was performed using 13 types of general foodborne bacteria, including Gram‐positive and ‐negative strains. As shown in Table 1, natto peptide exhibited a broad antimicrobial effect against both bacteria, and the minimum concentrations differed among different bacterial strains, with MICs ranging from 6 to 30 μg/ml.

**TABLE 1:** Antibacterial activity of the purified natto peptide

Bacterial species	MIC (μg/ml)
Gram‐positive	
*Bacillus subtilis* ATCC 6633	10
*Bacillus subtilis* AHU 1035	12
*Listeria monocytogenes* ATCC 221633	15
*Listeria monocytogenes* ATCC 19115	15
*Listeria monocytogenes* CMCC(B) 54002	15
*Staphylococcus aureus* ATCC 43300	20
*Staphylococcus aureus* ATCC 25923	20
Gram‐negative	
*Escherichia coli* ATCC 25922	6
*Escherichia coli* ATCC 8739	6
*Escherichia coli* O157:H7 ATCC 35150	6
*Enterobacter sakazakii* ATCC 29544	10
*Salmonella typhimurium* ATCC 14028	30
*Pseudomonas aeruginosa* ATCC 902	25

#### 3.1.2 Time‐killing curve of natto peptide

**FIGURE 1:**
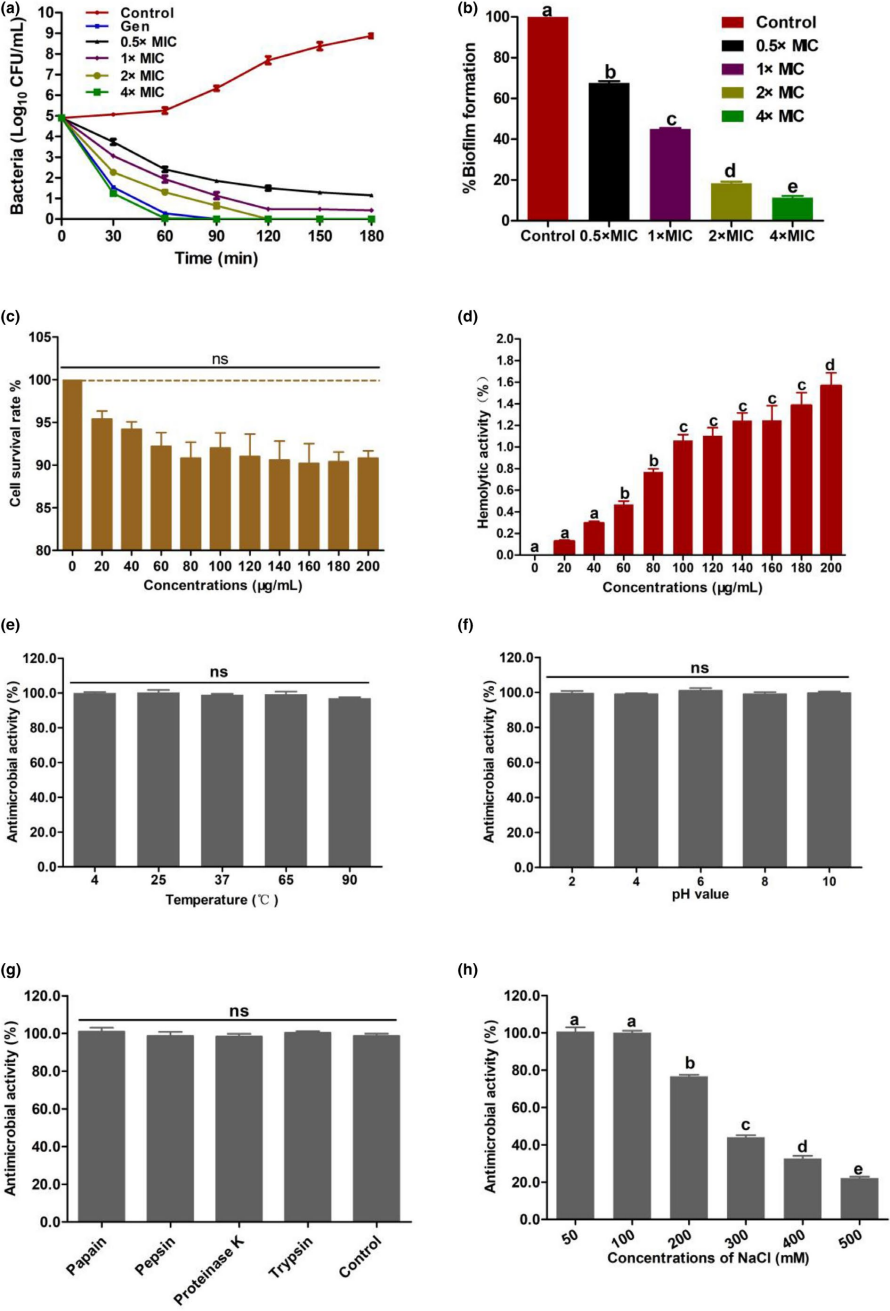
Antimicrobial property of natto peptide. (a) Time‐killing kinetic curve of purified natto peptide. (b) Biofilm formation inhibition curve. (c) Survival rate of cells analyzed through the methyl‐thiazol‐diphenyltetrazolium (MTT) assay method. (d) Hemolytic effect of natto peptide against rabbit erythrocytes. (e) Thermostability of natto peptide against *Escherichia* *coli*. (f) pH resistance of natto peptide against *E. coli*. (g) Saline resistance of natto peptide against *E. coli*. All the data are shown as the mean ± SD of triplicate measurements, *n* = 3. ns, nonsignificance. (h) Different superscript lowercase letters within each group represent significant difference (*p* < .05)

A time‐killing curve of natto peptide against *E. coli* O157 was plotted to determine the antimicrobial rate of natto peptide. As shown in Figure 1a, the bacterial counts (lg CFU/ml) of the control group were 8.7 after 3 h of incubation. On the contrary, the 0.5× and 1× MIC treatment groups showed an obvious inhibitory effect, with bacterial counts (lg CFU/ml) of 1.81 and 0.34, respectively. Moreover, the 2× and 4× MIC treatments completely sterilized the bacteria in 2 and 1 h, respectively. Furthermore, the killing curve of the gentamicin treatment group exhibited a similar tendency with the 4× MIC treatment group, and the bacteria were completely killed in 1.5 h.

#### 3.1.3 Antibiofilm properties of natto peptide

As shown in Figure 1b, the natto peptide treatment groups displayed a significant decrease (*p* < .0001) compared with control group on the formation of biofilm. At various concentrations (0.5×, 1×, 2×, and 4× MICs) of natto peptide, the biofilm formation of *E. coli* O157 decreased to 67%, 43%, 19%, and 11%, respectively.

#### 3.1.4 Cell cytotoxicity and hemolytic activity of natto peptide

As shown in Figure 1c, the cell survival rate at various concentrations of natto peptide had no significant difference (*p* > .05), compared with that of the control. Moreover, the hemolytic activity of natto peptide was <1.8% even at a concentration of 200 μg/ml (Figure 1d), which showed nearly no hemolytic effect.

#### 3.1.5 Stability of natto peptide

The stability of natto peptide in extreme external conditions, including thermostability, acid–base, and proteinase and saline resistance, was determined. As shown in Figure 1e–g, the antimicrobial activity of natto peptide was not affected in different temperatures (4–90°C; *p* > .05), pH values (*p* > .05), and protease digestions (*p* > .05). However, it was impaired with the increase in the concentration of NaCl (Figure 1h; *p* < .0001), indicating that the natto peptide was not resistant to high‐concentration saline.

#### 3.1.6 Antioxidant activity of natto peptide

**FIGURE 2:**
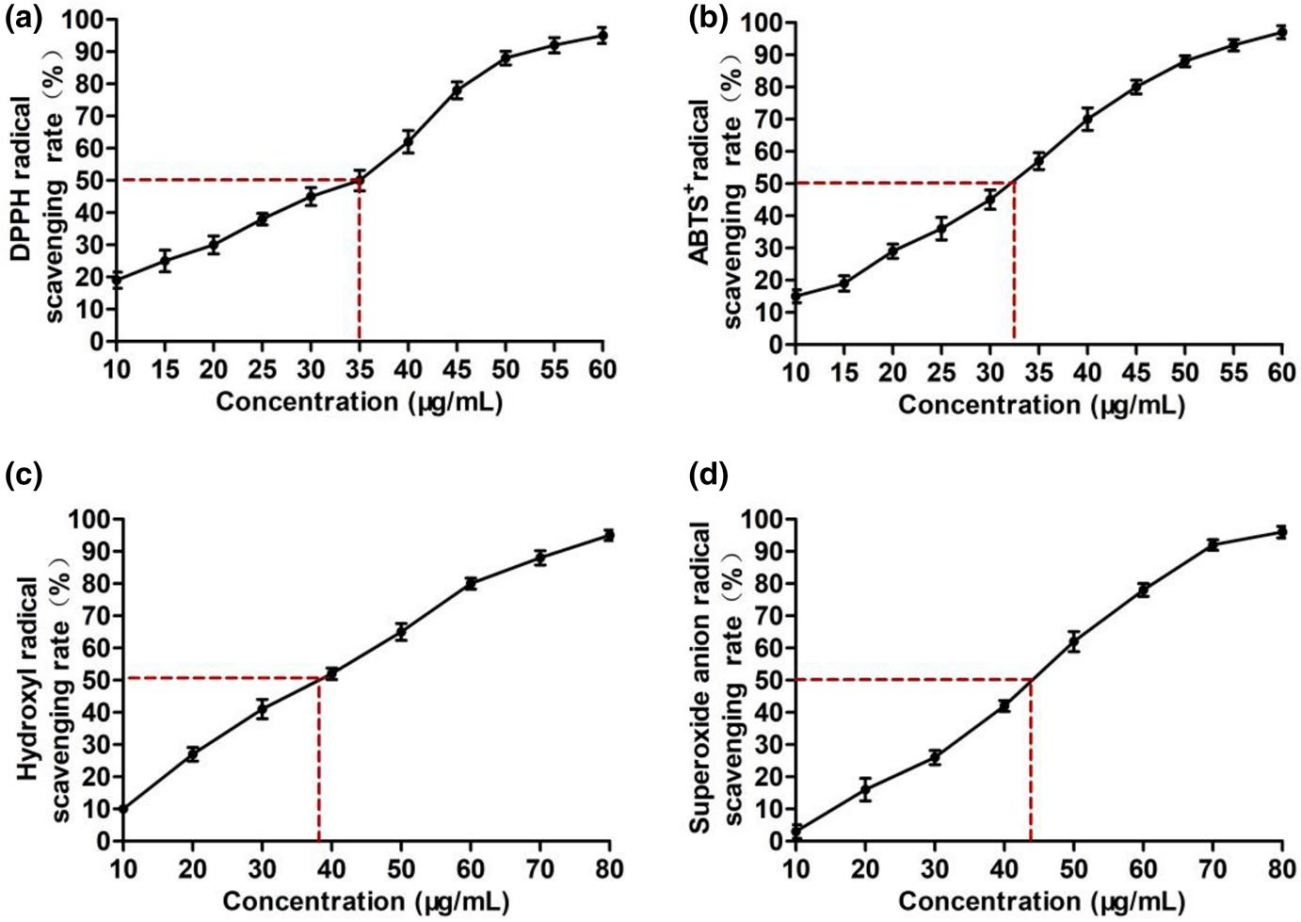
Antioxidant activity of natto peptide. (a) 2,2′‐diphenyl‐1‐picrylhydrazyl (DPPH) radical scavenging assay. (b) 2,2′‐azino‐bis(3‐ethylbenzothiazoline‐6‐sulfonic acid) (ABTS^+^) radical scavenging assay. (c) Hydroxyl radical scavenging assay. (d) Superoxide anion radical scavenging assay. All the data are shown as the mean ± SD of triplicate measurements

As shown in Figure 2a–d, the antioxidant effect of natto peptide was analyzed by detecting the scavenging ability of DPPH, ABTS^+^, hydroxyl and superoxide anion free radicals, with IC_50_ values of 35.0, 32.5, 39.2, and 42.5 μg/ml, respectively. The results suggested a dose‐dependent manner of antioxidant activity of natto peptide.

### 3.2 Application of natto peptide on pork during refrigerated storage

#### 3.2.1 Microbial growth analysis

**FIGURE 3:**
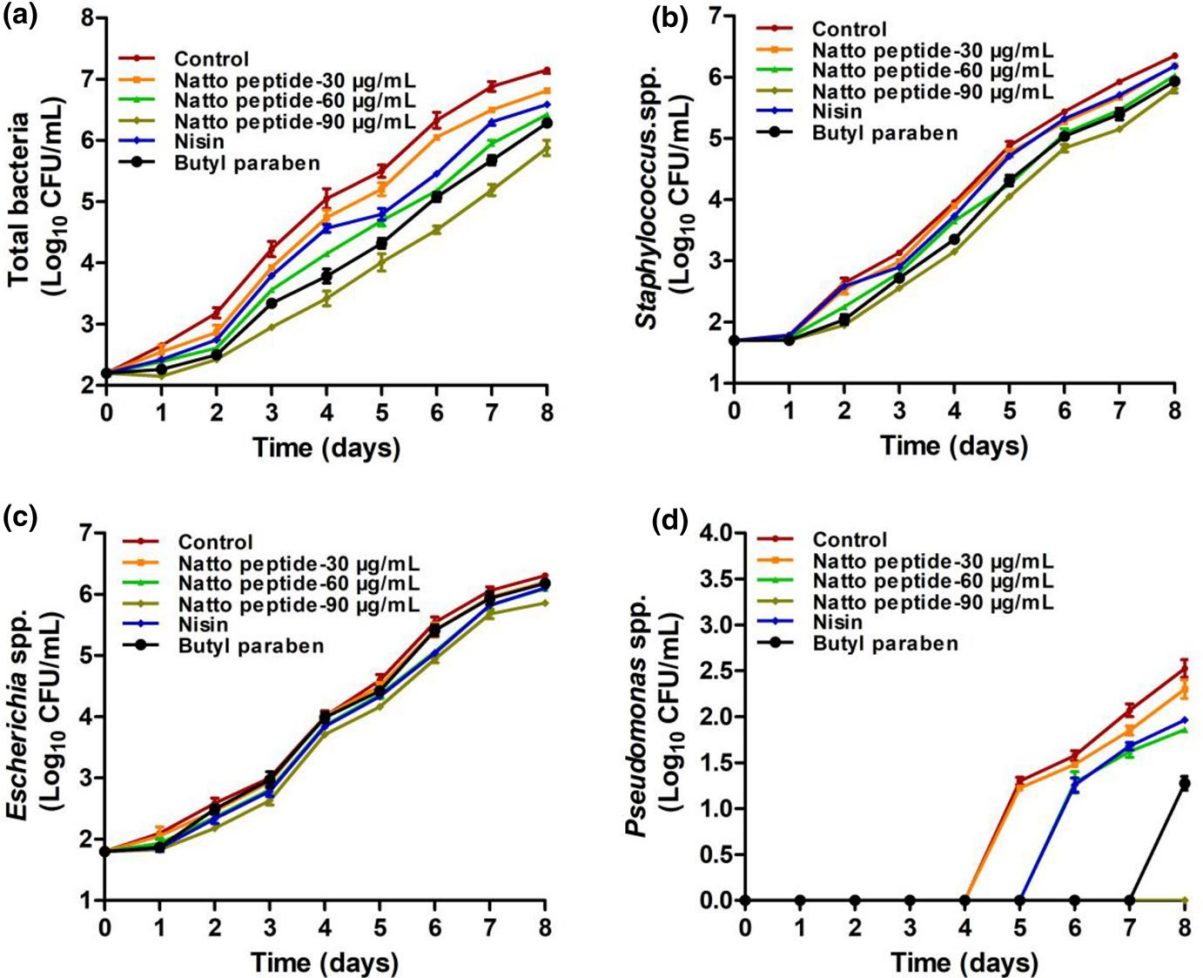
Microbial growth analysis of natto peptide used in pork storage. (a) Total bacterial growth of natto peptide treated during pork storage. (b) *Staphylococcus* spp. growth of natto peptide treated during pork storage. (c) *Escherichia* spp. growth of natto peptide treated during pork storage. (d) *Pseudomonas* spp. growth of natto peptide treated during pork storage

The antibacterial function of natto peptide during pork refrigerated storage was determined using the colony counting method, including the growth of total bacteria, *Staphylococcus* spp., *Escherichia* spp., and *Pseudomonas* spp. As shown in Figure 3a–d, the antibacterial effects of natto peptide treatment groups (30, 60, and 90 μg/ml) against total bacteria, *Staphylococcus* spp., *Escherichia* spp., and *Pseudomonas* spp., decreased significantly in a dose‐dependent manner compared with those of the control groups during the 8‐day storage. Moreover, the nisin treatment group showed a moderate inhibitory effect similar to that under 30 μg/ml of natto peptide. Furthermore, the butyl paraben treatment group showed an effective inhibition function similar to that under 90 μg/ml of natto peptide.

#### 3.2.2 Spoilage model test

*Staphylococcus* spp., *Escherichia* spp., and *Pseudomonas* spp. were used as artificial bacteria in pork to establish a spoilage model. As shown in Table 2, the counts of *E. coli* O157:H7 ATCC 35150 in pork during 8 days of storage were decreased (*p* < .05) by treatment with natto peptide, and the growth of bacteria was inhibited as the concentration of natto peptide increased compared with control. The growth of *S. aureus* ATCC 43300 in pork treated with natto peptide during storage was inhibited (*p* < .05) with a dose‐dependent manner. The counts of *P. aeruginosa* ATCC 902 increased slightly (*p* < .05), owing to the addition of natto peptide compared with control. Of note, the counts of three microorganisms treated with nisin showed no significant differences (*p* > .05) compared with 30 μg/ml of natto peptide treated groups, and the counts of three microorganisms treated with butyl paraben showed no significant differences (*p* > .05) compared with 90 μg/ml of natto peptide treated groups.

**TABLE 2:** Microbial challenge test of natto peptide

Strains	Days	Control	Bacterial counts (Log CFU/g)			Nisin	Butyl paraben
			Natto peptide				
			30 μg/ml	60 μg/ml	90 μg/ml		
*Escherichia* *coli* O157:H7 ATCC 35150	0	8.23 ± 0.22^a^	8.14 ± 0.15^a^	8.22 ± 0.11^a^	8.15 ± 0.16^a^	8.14 ± 0.24^a^	8.16 ± 0.18^a^
	1	8.78 ± 0.15^a^	6.71 ± 0.11^b^	5.07 ± 0.15^c^	4.05 ± 0.17^d^	7.03 ± 0.11^b^	4.53 ± 0.12^d^
	5	8.95 ± 0.14^a^	7.01 ± 0.15^a^	5.98 ± 021^b^	4.68 ± 0.26^c^	8.15 ± 0.27^a^	5.34 ± 0.19^b^
	8	9.12 ± 0.13^a^	7.66 ± 0.17^b^	6.31 ± 0.18^c^	5.83 ± 0.21^c^	7.79 ± 0.20^b^	6.16 ± 0.15^c^
*Staphylococcus* *aureus* ATCC 43300	0	7.15 ± 0.16^a^	7.27 ± 0.15^a^	7.17 ± 0.14^a^	7.14 ± 0.19^a^	7.18 ± 0.21^a^	7.22 ± 0.18^a^
	1	7.68 ± 0.10^a^	6.81 ± 0.16^b^	6.07 ± 0.21^c^	5.23 ± 0.17^d^	6.75 ± 0.15^b^	6.01 ± 0.18^c^
	5	7.95 ± 0.20^a^	7.12 ± 0.15^b^	6.89 ± 0.11^b^	6.13 ± 0.12^c^	7.06 ± 0.17^c^	6.87 ± 0.23^b^
	8	8.23 ± 0.21^a^	7.72 ± 0.21^a^	7.03 ± 0.20^b^	6.42 ± 0.17^c^	7.57 ± 0.16^a^	7.11 ± 0.14^b^
*Pseudomonas aeruginosa* ATCC 902	0	7.43 ± 0.13^a^	7.56 ± 0.14^a^	7.52 ± 0.18^a^	7.48 ± 0.13^a^	7.58 ± 0.14^a^	7.46 ± 0.15^a^
	1	7.84 ± 0.21^a^	6.92 ± 0.19^b^	6.34 ± 0.14^c^	5.62 ± 0.15^d^	7.02 ± 0.13^b^	7.03 ± 0.17^b^
	5	7.90 ± 0.20^a^	7.44 ± 0.14^b^	7.01 ± 0.17^c^	6.15 ± 0.16^d^	7.36 ± 0.19^b^	6.45 ± 0.12^d^
	8	7.98 ± 0.11^a^	7.78 ± 0.19^a^	7.39 ± 0.23^a^	7.23 ± 0.24^b^	7.63 ± 0.15^a^	7.24 ± 0.16^a^

Note

Values are mean ± standard deviation of three replicates, different lowercase letters mean significantly different in the same row (*p* < .05).

Abbreviations: Log CFU, logarithms of colony‐forming units per gram.

#### 3.2.3 pH and drip loss

**FIGURE 4:**
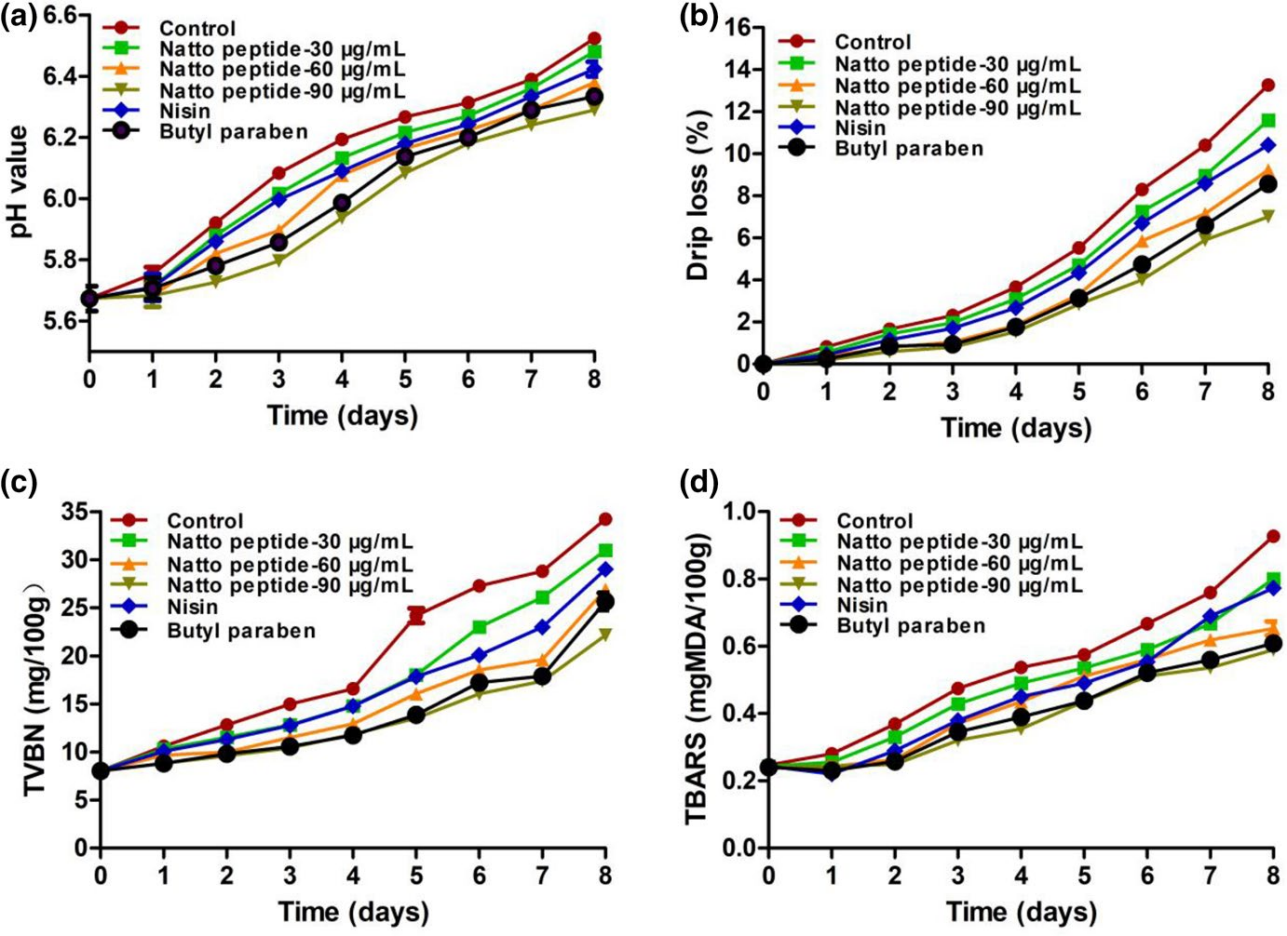
Freshness and physicochemical quality of pork during storage. (a) pH value of natto peptide treated during pork storage. (b) Drip loss of natto peptide treated during pork storage. (c) Total volatile basic nitrogen (TVB‐N) content of natto peptide treated during pork storage. (d) Thiobarbituric acid‐reactive substances (TBARS) content of natto peptide treated during pork storage

The pH variation of pork during storage is shown in Figure 4a. The natto peptide and butyl paraben treatment groups inhibited the increase in pH compared with the control group. However, the pork treated with nisin showed an obvious increase in pH during storage similar to the control group. In addition, the drip loss in all treatment groups increased with similar tendency, as shown in Figure 4b. A notable detail is that the 90 μg/ml of natto peptide showed the lowest increase among all groups during storage.

#### 3.2.4 TVB‐N

As shown in Figure 4c, the TVB‐N of all treatment groups decreased compared with that of the control group. The TVB‐N values of the natto peptide treatment groups (30, 60, and 90 μg/ml) on day 8 were 30.0, 25.2, and 19.4, respectively. The nisin and butyl paraben treatment groups also showed an inhibitory effect on TVB‐N, with concentrations of 28.1 and 26.0, respectively.

#### 3.2.5 TBARS

TBARS was related to the oxidation of pork during storage, as determined by measuring the content of MDA. As shown in Figure 4d, the TBARSs of all treatment groups increased with the extension of storage time, and 90 μg/ml of natto peptide extensively inhibited the increase in MDA. Moreover, the nisin and butyl paraben treatment groups showed an inhibitory effect on TBARS, with 0.5 and 0.6 mg/100 g on day 8, respectively.

#### 3.2.6 Sensory quality

The quality changes in pork acceptance during storage were determined by sensory evaluation. As shown in Table 3, the sensory scores of pork significantly reduced with the extension of storage time in the control group, whose sensory scores were <4 on day 4. On the contrary, the sensory scores of natto peptide treatment groups were decreased slightly on day 4 (*p* < .05), which showed significant differences compared with control. According to the evaluation criteria, various concentrations (30, 60, and 90 μg/ml) of natto peptide treated groups maintained the sensory scores of pork to acceptable (>5) at days 5, 6, and 7, respectively. In addition, the sensory scores of nisin and butyl paraben treated groups decreased slightly (*p* < .05) compared with control group during storage and showed no difference (*p* > .05) compared with 30 and 90 μg/ml of natto peptide treated groups.

**TABLE 3:** Sensory scores of the pork with different treatments

Days	Control	Treatment groups			Nisin	Butyl paraben
		Natto peptide				
		30 μg/ml	60 μg/ml	90 μg/ml		
0	9.76 ± 0.21^a^	9.76 ± 0.21^a^	9.76 ± 0.21^a^	9.76 ± 0.21^a^	9.76 ± 0.21^a^	9.76 ± 0.21^a^
1	8.23 ± 0.35^a^	9.02 ± 0.24^b^	9.24 ± 0.18^b^	9.26 ± 0.32^b^	9.12 ± 0.27^b^	9.16 ± 0.29^b^
2	7.11 ± 0.26^a^	8.65 ± 0.25^b^	8.93 ± 0.31^b^	9.02 ± 0.33^b^	8.75 ± 0.35^b^	8.93 ± 0.18^b^
3	4.89 ± 0.25^a^	6.71 ± 0.19^b^	7.87 ± 0.27^c^	8.14 ± 0.24^d^	6.89 ± 0.28^b^	8.01 ± 0.16^d^
4	3.87 ± 0.34^a^	5.38 ± 0.37^b^	6.98 ± 0.28^b^	7.34 ± 0.37^c^	5.54 ± 0.31^b^	7.13 ± 0.36^c^
5	2.79 ± 0.33^a^	4.23 ± 0.24^b^	5.84 ± 0.36^c^	6.01 ± 0.28^c^	4.13 ± 0.23^b^	5.89 ± 0.29^c^
6	2.02 ± 0.36^a^	3.91 ± 0.16^b^	4.17 ± 0.15^b^	5.21 ± 0.24^c^	3.96 ± 0.17^b^	5.05 ± 0.32^c^
7	1.65 ± 0.27^a^	2.84 ± 0.24^b^	3.82 ± 0.28^c^	4.31 ± 0.32^d^	2.90 ± 0.19^b^	4.03 ± 0.25^c^
8	0.64 ± 0.27^a^	1.68 ± 0.23^b^	1.72 ± 0.34^b^	2.24 ± 0.25^c^	1.89 ± 0.20^b^	2.01 ± 0.28^c^

Note

Values are mean ± standard deviation of three replicates, different lowercase letters mean significantly different in the same row (*p* < .05).

## 4 DISCUSSION

Foodborne spoilage bacteria causing food safety problems could seriously harm human health, and they have attracted increasing attention all over the world. AMPs have been accepted as a novel promising biopreservative in the food antiseptic field for their broad antibacterial spectrum, efficient bactericidal capability, and low cytotoxicity compared with chemical preservatives. Although some AMPs, such as nisin (Gharsallaoui et al., 2016), As‐CATH4 (Guo et al., 2017), As‐CATH5 (Guo et al., 2017), and HcCATH (Yu et al., 2017), have been identified and demonstrated potential applicability in various kinds of foods, the preparation costs, antiseptic efficiency, and mechanisms need to be further reduced, improved, and investigated, respectively. The *P. pastoris*‐derived natto peptide showed a broad antibacterial spectrum, including Gram‐positive and ‐negative strains, as shown in Table 1. However, the naturally obtained natto peptide only presented a narrow antibacterial activity against *S. pneumoniae* and *B. subtilis* groups (Kitagawa et al., 2017). The time‐killing curve indicated that natto peptide could sterilize *E. coli* in 90 min, suggesting that it possesses bacteriostatic and bactericidal activities. Meanwhile, the biofilm formation was related to the drug resistance of bacteria (De Zoysa et al., 2015). The results showed that 1× MIC of natto peptide inhibited the biofilm formation below 50%, and the antibacterial effect was dose dependent. Nevertheless, the detailed antimicrobial mechanism of natto peptide still needs to be investigated in further studies.

The applicability of natto peptide on different external conditions similar to foods, such as pork, during processing, transportation, and storage was investigated by determining the stability in extreme conditions, the cytotoxicity on mammalian cells, and hemolytic and antioxidant activities. The analysis results on the stability of natto peptide showed that high temperature, strong acid/base, and proteinase digestion treatment could not affect the antimicrobial activity of natto peptide, except for NaCl treatment, which attenuated the antimicrobial activity to lower than 50% when the NaCl concentration was increased from 200 to 500 mM. AMPs perform bactericidal activity by mutual attraction and attachment between the negatively charged appearances of bacteria and cationic sites of the peptides (Plant et al., 2010). Thus, the decreased antimicrobial activity of natto peptide under a high concentration of NaCl could be attributed to the competition between the peptides and Na^+^. Moreover, natto peptide showed a prominent antioxidant effect, indicating that it could be used as an antioxidant agent in pork storage to prevent the oxidation process that seriously deteriorates pork quality. All of these results implied that natto peptide could be potentially used as a pork preservative for processing, transportation, or storage in various external environments.

Bacterial count was used to analyze the inhibitory function of natto peptide to further evaluate its antiseptic properties against the typical foodborne microorganisms (*Staphylococcus* spp., *Escherichia* spp., and *Pseudomonas* spp.) in pork during refrigerated storage. Considering that bacterial growth is the main cause of spoilage, which leads to deterioration of quality and safety, TVC is an essential parameter to judge the antiseptic characters of preservatives. According to the results of total bacterial analyses, 90 μg/ml of natto peptide showed prominent antiseptic effect and decreased the total viable counts (TVCs) of microorganisms during pork refrigerated storage. It exerted a better antibacterial effect with less addition than 100 μg/ml of butyl paraben, which is a conventional chemical preservative accepted in food antiseptic field, with broad antimicrobial spectrum. These results were identical to those of some AMPs, such as housefly pupae peptide (Wang et al., 2010), mytichitin‐CB (Meng et al., 2021), Lactolisterin BU (Dong et al., 2021), and MccJ25(G12Y) (Corbalán et al., 2021), which have been investigated in pork chilled storage. *Pseudomonas* spp. and *Escherichia* spp. adversely affected the quality of pork during chilled storage, and they were too stubborn to be eliminated by conventional measures, including freezing, surface dehydration, and simulated spray chilling. The performance of nisin in the present study was barely satisfactory, which could be attributed to its poor antibacterial effect against Gram‐negative strains and the influence of phospholipids and glutathione that exist in pork constituents. However, the antibacterial function of natto peptide was impervious to the pork components, in agreement with its resistance to proteinase digestion and the other attributes of stability. Moreover, the results of the established pork spoilage model further confirmed the antimicrobial properties of natto peptide in pork storage. They were in agreement with the results of some AMPs, including bacaucin‐1 and pentocin 31‐1 (Liu et al., 2017; Zhang et al., 2010), which have been tested in a spoilage model.

The physicochemical indices of pork including microbial, TVB‐N, lipid oxidation, pH, drip loss, and sensory quality have been recognized as indispensable evaluation criteria for the freshness and safety of pork (Cao et al., 2019; Przybylski et al., 2016; Zhang et al., 2018). The inhibition of bacteria growth could reduce the production of volatile alkaline nitrogen molecules such as amine, which might be the reason why natto peptide decreased the pork pH during chilled storage (Cao et al., 2019; Ruan et al., 2019). The natto peptide dramatically decreased the drip loss of pork during storage compared with control group which was related to the maintenance of meat freshness. The concentration of TVB‐N in pork reflected its fresh content, which was produced mainly owing to proteolysis by the spoilage bacteria (Bekhit et al., 2021). Natto peptide treatment decreased the generation of TVB‐N and increased the storage days in a dose‐dependent manner compared with the control. Therein, high‐dose (90 μg/ml) natto peptide exhibited the lowest concentration of TVB‐N among the three treatment groups, including nisin, butyl paraben, and natto peptide. These results could be explained by the excellent bactericidal property of natto peptide, which was similar to those of some biopreservatives such as mytichitin‐CB (Meng et al., 2021), nisin (Gharsallaoui et al., 2016), and green tea aqueous extract (GTAE; Montaño‐Sánchez et al., 2020). Furthermore, the lipid oxidation‐formed byproducts, including aldehydes and other fetid decomposition products, during pork storage could disrupt the sensory qualities and lead to spoilage (Xiong et al., 2020). The results of TBARS assay showed that natto peptide also decreased the generation of TBARS in pork. However, nisin and butyl paraben displayed no significant difference in TBARS with control, which could be attributed to the antioxidant function of natto peptide investigated in this study. Fasseas group confirmed that oregano oil could be used as an antioxidant to inhibit the fat oxidation during meat refrigeration, which had antibacterial and antioxidant activity in vitro (Fasseas et al., 2008). Previous studies have elucidated that AMPs with scavenging capacities of free radicals were also conducive to the prevention of lipid oxidation, indicating that natto peptide could be used as an antioxidant agent to help pork retain its freshness (Meng et al., 2021). The sensory quality of pork is a critical factor for the acceptance of consumers. The microbial growth, lipid oxidation, pH variation, and drip loss during chilled storage would affect the sensory quality of pork (Siripatrawan & Noipha, 2012). The sensory evaluation results were consistent with those of physicochemical analyses as expected, that is, natto peptide extended the shelf life of pork and delayed the occurrence of deterioration during pork refrigerated storage.

## 5 CONCLUSIONS

This study indicated that the natto peptide prepared from *P. pastoris* by the DNA recombinant technology exhibited a broader antibacterial spectrum, including Gram‐positive and ‐negative strains, and enhanced bactericidal property, with MICs ranging from 6 to 30 μg/ml. The addition of natto peptide during fresh‐pork refrigerated storage predominantly decreased the growth of *Staphylococcus* spp., *Escherichia* spp., and *Pseudomonas* spp. In addition, the bactericidal properties remained in force when the natto peptide was used in pork models artificially contaminated with bacteria. Moreover, the application of natto peptide (90 μg/ml) significantly inhibited the increase in pH variation and drip loss, decreased the generation of TVB‐N and TBARS, and maintained a high sensory quality score during pork storage at 4°C. These results implied that *P. pastoris‐*derived natto peptide could significantly extend the shelf life of pork, and it has a potential to be a promising antiseptic biopreservative to replace chemical preservatives during pork refrigerated storage.

## CONFLICTS OF INTEREST

The authors declare no conflict of interest.

## AUTHOR CONTRIBUTIONS

**Bin Dong:** Conceptualization (equal); Funding acquisition (equal); Project administration (equal); Supervision (equal); Writing – original draft (equal). **Cailing Yu:** Data curation (equal); Investigation (equal). **Yanjun Lin:** Data curation (equal); Investigation (equal). **Guowen Zhou:** Conceptualization (equal); Data curation (equal); Formal analysis (equal). **Chunlong Sun:** Funding acquisition (equal); Software (equal); Writing – review & editing (equal). **Jun Wang:** Funding acquisition (equal); Resources (equal); Software (equal). **Tao Wu:** Funding acquisition (equal); Resources (equal); Software (equal).

## Supporting information

Supplementary Material

## Data Availability

Data available on request from the authors.
