# Supplementary material for: Antimicrobial property of Pichia pastoris‐derived natto peptide against foodborne bacteria and its preservative potential to maintain pork quality during refrigerated storage
Source: Food Sci Nutr. 2022 Jan 8;10(3):914–25. doi: 10.1002/fsn3.2722 (PMC8907714; doi:10.1002/fsn3.2722)
Supplement: Supplementary file 1 — Supplementary Material [file FSN3-10-914-s001.docx]

**Supplementary materials:**

***1. Materials and methods***

***1.1. Production of natto peptide from P. pastoris***

***1.1.1. Vector construction and positive-transformant screening***

The yeast codon optimized DNA encoding sequences of natto peptide with a 6× His tag at the N terminal were synthesized and sequenced by Genewiz Company (Suzhou, China) and then subcloned into pPICZα-A plasimid over *Eco*RI and *Kpn*I restriction enzyme sites, generating the pPICZα-A-natto peptide vector. The constructed vectors were transformed into *P. pastoris* X-33 cells by electroporation as described previously (Meng, et al., 2016), and the positive transformed colonies were screened out by Zeocin antibiotics (150 μg/mL) and then identified and confirmed by colony PCR method.

***1.1.2. Expression and shake-flask cultivation optimization***

The confirmed positive yeast colonies were initially inoculated in BMGY medium at 28 °C and 220 rpm/min for 24 h. The cell cultures were further transferred in BMGY medium at a ratio of 5% and incubated until the OD_600_ value reached 8–10 before the cells were harvested and washed by sterile distilled water. Then, the washed cells were cultured in an equal volume of BMMY medium for 144 h of continuous induction. Meanwhile, a final concentration of 1.0% (v/v) methanol in the culture suspensions was achieved by adding 0.22 μm-filtered 100% methanol every 24 h. Moreover, 1 mL of the supernatants of the culture was harvested every 24 h to analyze the expression by Tricine-SDS-PAGE and silver staining. The supernatants of the induction culture were obtained every 24 h to optimize the methanol induction time for natto peptide yield, with a final concentration of methanol at 1.0% (v/v). For optimization of the methanol concentration for natto peptide yield, various final concentrations (0.50%, 0.75%, 1.00%, 1.25%, 1.50%, 1.75%, and 2.00%) of methanol were used to determine the secreted amount of natto peptide, and supernatant samples were collected and analyzed after 120 h of induction.

***1.1.3. Purification of natto peptide from supernatant of yeast culture***

Purification of natto peptide from the supernatant of yeast culture was performed using a Ni-NTA column as described previously (Dong, et al., 2020). In brief, the supernatants of the induction culture were harvested by centrifugation at 4 °C at a speed of 12,500 rpm/min for 30 min after 144 h of induction with 1.0% methanol. The obtained supernatants were initially dialyzed with Ni-NTA column binding buffer (20 mM NaH_2_PO_4_, 500 mM NaCl, and 5 mM imidazole; pH 7.4) for 24 h before equilibrating with Ni-NTA column at 4 °C overnight. Then, the protein-bounded column was washed by 3–5 column volumes of washing buffer (20 mM NaH_2_PO_4_, 500 mM NaCl, and 60 mM imidazole; pH 7.4) to remove the unbound proteins. Finally, the purified natto peptide was acquired using elution buffer (20 mM NaH_2_PO_4_, 500 mM NaCl, and 500 mM imidazole; pH 7.4) with a high concentration of imidazole and then stored at −80 °C for the subsequent experiments after freeze drying.

**2. Results**

***2.1. Production of natto peptide from P. pastoris***

***2.1.1. Construction of expression vector and transformation into P. pastoris***

The natto peptide encoding sequence with an *Eco*RI and *Kpn*I at the 5′ and 3′ ends, respectively, was synthesized and subcloned into pPICZα-A plasimid over *Eco*RI and *Kpn*I restriction enzyme sites, generating the pPICZα-A-natto peptide vector (Fig. S1A). In the expression cassette, the natto peptide encoding fragment was attached to the 3′ end of the α-factor secretion signal, downstream of the alcohol oxidase I (AOX1) promoter. After the linearized vector was electroporated into *P. pastoris* X-33, 15 zeocin-resistant colonies were identified by colony PCR. All of those colonies showed specific bands, as shown in Fig. S1B, indicating that the natto peptide encoding sequences were inserted into the genome of *P. pastoris*.

***2.2.2. Shake-flask cultivation optimization***

The induction duration and methanol concentration were optimized to improve the yield of natto peptide in shake-flask cultivation. As shown by Tricine-SDS-PAGE in Figs. S1C and D, the secreted natto peptide increased with the extension of induction time from 0 h to 144 h [methanol concentration (v/v) =1.0%]. After 96 h of induction, the yield of natto peptide did not improve significantly. For the optimization of methanol concentration, various concentrations (0.50%, 0.75%, 1.00%, 1.25%, 1.50%, 1.75%, and 2.00%) of methanol were applied to determine the highest level of natto peptide secreted in the culture supernatant. As shown in Figs. S1C and D, with a final concentration of methanol at 1.0% (v/v) and 96 h induction.

***2.2.3. Purification of natto peptide***

The natto peptide in *P. pastoris* cells was cultured under optimized parameters to purify it from the supernatant of cell cultures (1.0% of methanol and 96 h of induction duration). After the culture supernatant was purified by Ni-NTA column and imidazole was removed by dialysis, the isolated natto peptide was analyzed by Tricine-SDS-PAGE assay, which showed a single band with a molecular weight of 6.5 kDa as expected (Fig. S1E) with 95.0% purity.

***3. Discussion***

Shinichi Yokota’s group recently isolated natto peptide directly from *B. subtilis-*fermented Natto and characterized its antibacterial properties and mechanism (Kitagawa et al., 2017). Considering the preparation costs and to further improve the antimicrobial activity, the *P. pastoris* system was used in the present study as host to overexpress natto peptide for the first time and optimize the induction time and methanol concentration to obtain optimal yield. Unexpectedly, the yeast-derived natto peptide showed a broad antibacterial spectrum, including Gram-positive and -negative strains, as shown in Table 1. However, the naturally obtained natto peptide only presented a narrow antibacterial activity against *S. pneumoniae* and *B. subtilis* groups (Kitagawa et al., 2017). *P. pastoris* has been used as cell factory to produce many AMPs that originated from various sources, including plants, animals, and microorganisms, due to the eukaryotic post-translational modifications in yeast cells, including protein proper folding and glycation. Some yeast-derived AMPs exerted more enhanced antimicrobial activity and broader antimicrobial scopes than their original peptides (Karbalaei et al., 2020). Similarly, eukaryotic cell factories, such as *Chlamydomonas reinhardtii*, strengthened and increased the antimicrobial activities and microorganism types of AMPs, including Mytichitin-A (Dong et al., 2018), LS2 (Liu et al., 2020), and ToAMP4 (Xue et al., 2020). According to the structure prediction model, natto peptide is rich in α-helix, and it showed a similar character with cathelicidin family, which is a classical AMP with broad antimicrobial spectrum, including Gram-positive and -negative strains. Moreover, natural natto peptide was extracted from the Natto fermented by *B. subtilis*. The possible reasons for the differences in the antimicrobial performance for the two derived peptides are that natural natto peptide derived from a prokaryotic cell, and it lacks essential modifications and processing compared with translational peptides.

**References:**

Dong, B., Cheng, R. Q., Liu, Q. Y., Wang, J., & Fan, Z. C. (2018). Multimer of the antimicrobial peptide Mytichitin-A expressed in Chlamydomonas reinhardtii exerts a broader antibacterial spectrum and increased potency. *J Biosci Bioeng, 125*(2), 175-179.

Karbalaei, M., Rezaee, S. A., & Farsiani, H. (2020). Pichia pastoris: A highly successful expression system for optimal synthesis of heterologous proteins. *J Cell Physiol, 235*(9), 5867-5881.

Kitagawa, M., Shiraishi, T., Yamamoto, S., Kutomi, R., Ohkoshi, Y., Sato, T., Wakui, H., Itoh, H., Miyamoto, A., & Yokota, S. I. (2017). Novel antimicrobial activities of a peptide derived from a Japanese soybean fermented food, Natto, against Streptococcus pneumoniae and Bacillus subtilis group strains. *AMB Express, 7*(1), 127.

Liu, Y. X., Li, Z. F., Lv, Y. J., Dong, B., & Fan, Z. C. (2020). Chlamydomonas reinhardtii-expressed Multimer of Bacteriocin LS2 Potently Inhibits the Growth of Bacteria. *Process Biochemistry, 95*.

Meng D M , Dai H X , Gao X F , Zhao JF, Ling X, Dong B, Zhang ZQ, & Fan ZC. (2016) Expression, purification and initial characterization of a novel recombinant antimicrobial peptide Mytichitin-A in Pichia pastoris. *Protein Expres & Purif*, 127:35-43.

**Supplementary data:**

**Fig.S1
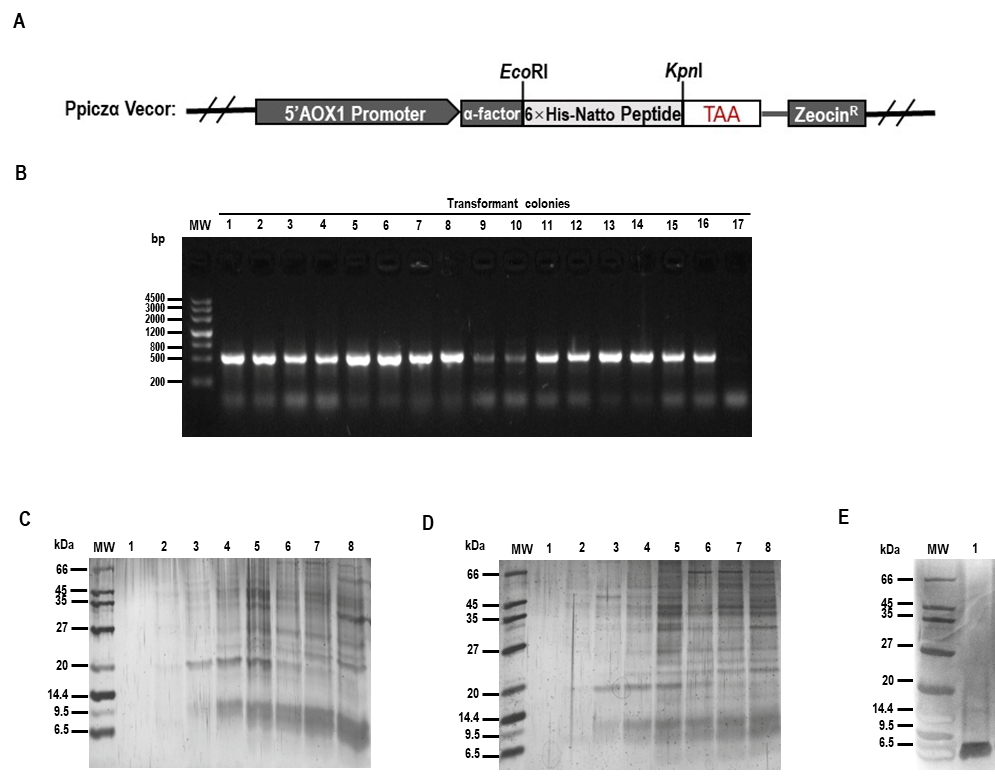
** **Production of natto peptide from *P. pastoris*.** (A) The scheme of the pPICZα-natto peptide. (B) Identification of positive transformant. Lane MW: DNA Marker III. Lane 1: pPICZaA-natto peptide positive control; Lanes 2-16: 15 Zeocin-resistant colonies; Lane 17: *P. pastoris* X -33 cell containing pPICZaA as a negative control. (C) Expression of natto peptide in the supernatant from the positive *P. pastoris* colony and optimization of induction duration. Lane MW: Protein Molecular weight ladder; Lane1: supernatant of *P. pastoris* X -33 cell containing pPICZaA as a negative control; Lane 2-8: supernatant of positive transformant taken at 0, 24, 48, 72, 96, 120, 144 h of induction, respectively. (D) Expression of natto peptide in the supernatant from the positive *P. pastoris* colony and optimization of methanol concentration. Lane MW: Protein Molecular weight ladder; Lane1: supernatant of *P. pastoris* X-33 cell containing pPICZα-A as a negative control; Lane 2-8: supernatant of positive transformant taken at 144 h of induction with methanol concentration at 0.50%, 0.75%, 1.00%, 1.25%, 1.50%, 1.75%, and 2.00%, respectively. (E) Tricine-SDS-PAGE analysis of the purified natto peptide from the supernatant of the positive *P. pastoris* colony.
